# Feasibility and Acceptability of Just Breathe, A Novel Handheld Mindful Breathing Device, for Postpartum Stress: Pilot, Single-Arm Pre-Post Study

**DOI:** 10.2196/85321

**Published:** 2026-02-27

**Authors:** Sarai K Sales, Stephanie D Nunez, Lindsay Spratt, Tracy L Jackson, Janet L Rousseau, Crystal F Ware, Kristopher L Britton, Margaret H Bublitz, Nina K Ayala, Adam K Lewkowitz

**Affiliations:** 1 Department of Obstetrics & Gynecology Warren Alpert Medical School Brown University Providence, RI United States; 2 Brown School of Public Health Brown University Providence, RI United States; 3 Department of Psychiatry and Human Behavior Warren Alpert Medical School Brown University Providence, RI United States

**Keywords:** postpartum stress, postpartum anxiety, heart rate variability, digital health, mindful breathing

## Abstract

Among newly postpartum people, the novel Just Breathe guided breathing device showed high user satisfaction scores and self-perceived reductions in stress, although the Cohen d effect on heart rate variability and stress or anxiety symptoms as measured by validated psychometric scales was minimal.

## Introduction

Over 1 in 5 birthing people are diagnosed with perinatal mood or anxiety disorders, and many who do not meet diagnostic thresholds for these conditions experience stress or anxiety that negatively impacts infant bonding or decrease breastfeeding rates [1-3]. Mindful breathing can improve mood and stress symptoms and increase heart rate variability (HRV) [4]. Defined as the beat-to-beat variability in heart rate, HRV is inversely associated with symptoms of depression and stress and can be assessed using wrist monitors [5]. A novel device, Just Breathe, incorporates airflow-guided cues that optimize adherence to evidence-based mindful breathing patterns shown to reduce stress symptoms [6]. Just Breathe is lifted to the user’s slightly opened lips, and it directs gentle airflow into the mouth to prompt inhalation and on the upper lip to cue exhalation over a 3-minute cycle (Figure S1 in Multimedia Appendix 1); it is yet to be examined in clinical settings. Our primary aim was to assess Just Breathe’s feasibility, acceptability, and perceived effectiveness among newly postpartum people. Our secondary, exploratory aim was to explore its effects on HRV, anxiety symptoms, and perceived stress.

## Methods

Participants were recruited at a tertiary care women’s hospital. Eligible patients were English-speaking, ≥18 years old, and admitted to the postpartum unit during their childbirth hospitalization. Patients were excluded if incarcerated or if their infant required neonatal intensive care unit (NICU) admission. After providing consent, participants completed demographic surveys and the Perceived Stress Scale-4 (PSS-4) [7] and Generalized Anxiety Disorder-7 (GAD-7) [8] scale to assess for symptoms of stress and anxiety, respectively. A research staff member applied a Kairos Biostrap wrist monitor, a commercially available high-grade wearable that uses infrared photoplethysmography to assess HRV, measured in root mean square of successive differences. The wrist strap monitor was connected to a smartphone app, Vital Science, which summarizes HRV during the 3-minute cycle. After the participant rested in her hospital bed or chair for 5 minutes without interruption, one Vital Science cycle was conducted before Just Breathe use, and one Vital Science cycle was conducted while the participant used Just Breathe for 3 minutes. Participants were provided a Just Breathe device and encouraged to use it at least 3 times for up to 24 hours. The day after consent, a research staff member repeated the same procedures to assess HRV.

Participants then completed PSS-4, GAD-7, system usability scale (SUS; assesses patient experience with ease of use), Client Satisfaction Questionnaire (CSQ; assesses patient satisfaction with device), and the Decisional Regret Scale (assesses regret after making a health-related decision). To preserve validity and reliability of the surveys while ensuring they pertained to Just Breathe, SUS and CSQ were minimally modified by replacing the words “system” and “service,” respectively, with the words “Just Breathe device.” Descriptive statistics (means, SD, and percentages) compared initial and final HRV assessments and psychometric survey results. For HRV, GAD-7, and PSS-4, effect sizes for pre-post changes were calculated using paired Cohen *d*. Hypothesis testing was not performed due to the sample size. This study was approved by the institutional review board at Women and Infant Hospital of Rhode Island before study initiation (WIH 24-0049; approved on October 14, 2024). Informed consent was obtained, all data were de-identified, and participants could opt out per their preference. Those who completed the study received a US $5 gift card.

## Results

Among 46 consented participants, 40 completed all survey measures and were included. Participants were racially, ethnically, and socioeconomically diverse: 48% (19/40) were non-White or Hispanic and 40% (16/40) had federal health insurance. Among the 40 study participants, 83% (n=33) were mostly or very satisfied with Just Breathe, and most (n=30, 75%) believed the device met their needs. In terms of perceived effectiveness, 88% (n=35) felt that Just Breathe helped improve their symptoms of stress or anxiety, and 85% (n=34) would recommend the device to a friend who was pregnant or newly delivered (Table 1).

Regarding exploratory analyses on psychometric outcomes (Table 2), although Just Breathe use was associated with a 25% increase in mean HRV, Cohen *d* analyses suggested a minimal effect of Just Breathe on HRV, GAD-7 scores, and PSS-4 scores.

**Table 1:** Participant outcomes: patient-reported questions (N=40).

Question			Value, n (%)
**How satisfied are you with the amount of information Just Breathe provided?**			
	Dissatisfied/indifferent	7 (17)	
	Mostly/very satisfied	33 (83)	
**To what extent has Just Breathe met your needs?**			
	Only a few/no needs met	10 (25)	
	Most/all needs met	30 (75)	
Did Just Breathe help you to deal more effectively with symptoms of stress or anxiety? Answer: Yes			35 (88)
If a friend were pregnant or newly delivered, would you recommend Just Breathe? Answer: Yes			34 (85)
If you were able to, would you use Just Breathe again? Answer: Yes			29 (73)

**Table 2:** Participants’ psychometric outcomes (N=40).

Measure	Before Just Breathe, mean (SD)	After Just Breathe, mean (SD)	Change pre/post Just Breathe^a^ (%)	Paired Cohen *d*
Heart rate variability (ms)	36.0 (19)	39 (18)	26	0.21
Perceived Stress Score-4	7.9 (2.3)	7.4 (2.5)	–8	–0.18
Generalized Anxiety Disorder-7	4.3 (3.7)	4.7 (3.8)	0.4	0.21

^a^For the pre/post change, no absolute value could be calculated, as only the change in mean (SD) was evaluated.

## Discussion

Among newly postpartum people, Just Breathe was feasible and acceptable as a stress- and anxiety-reduction intervention, with high satisfaction ratings and strong user perception of benefit. Our exploratory analyses showed a minimal effect on HRV or psychometric outcomes after using Just Breathe for less than 24 hours. This could be because the device had no measurable effect or due to study methodology. Specifically, the study occurred in the early postpartum period over a 24-hour time period. Postpartum hospitalization is characterized by nursing- and infant-derived sleep interruption, delivery-related pain, and hormonal fluctuations that may increase stress and anxiety, thereby reducing Just Breathe’s effect. Prior studies that demonstrated an association between mindful breathing and improved postpartum mood or stress symptoms required participants to use mindful breathing for up to 8 weeks [9,10]. Thus, the lack of a strong signal in our psychometric outcomes may be due to a short study period within a backdrop of profound physiologic and psychologic adjustments. This study’s strengths include using both physiological and psychological measures, high participant retention, and inclusion of a racially, ethnically, and socioeconomically diverse study population. Limitations include the single-arm design, small sample size, short study duration, and exclusion of non-English speaking people and those whose infants were in the NICU, who are at higher rates of having stress or anxiety symptoms. Just Breathe is a novel device that is acceptable and viewed as effective. We did not assess the frequency that participants used Just Breathe between enrollment and study conclusion, which can be considered a major limitation that precludes the analysis of a dose-response relationship, thereby limiting the conclusions that could be drawn on Just Breathe’s effect. In conclusion, a randomized controlled trial using Just Breathe as a study intervention for weeks, if not months, is needed to more accurately determine whether this promising device can improve perinatal physiologic regulation and symptoms of stress and anxiety after childbirth.

## Appendix

Multimedia Appendix 1 Diagram of the Just Breathe device mouthpiece and airflow haptic system.

## Abbreviations

CSQ: Client Satisfaction Questionnaire
GAD-7: Generalized Anxiety Disorder-7
HRV: heart rate variability
NICU: neonatal intensive care unit
PSS-4: Perceived Stress Scale-4
SUS: system usability scale
