# Supplementary figures and images for: Feasibility and Acceptability of Just Breathe, A Novel Handheld Mindful Breathing Device, for Postpartum Stress: Pilot, Single-Arm Pre-Post Study
Source: JMIR Form Res. 2026 Feb 27;10:e85321. doi: 10.2196/85321 (PMC12988350; doi:10.2196/85321)

**Figure S1.** Diagram of the Just Breathe device mouthpiece and airflow haptic system.


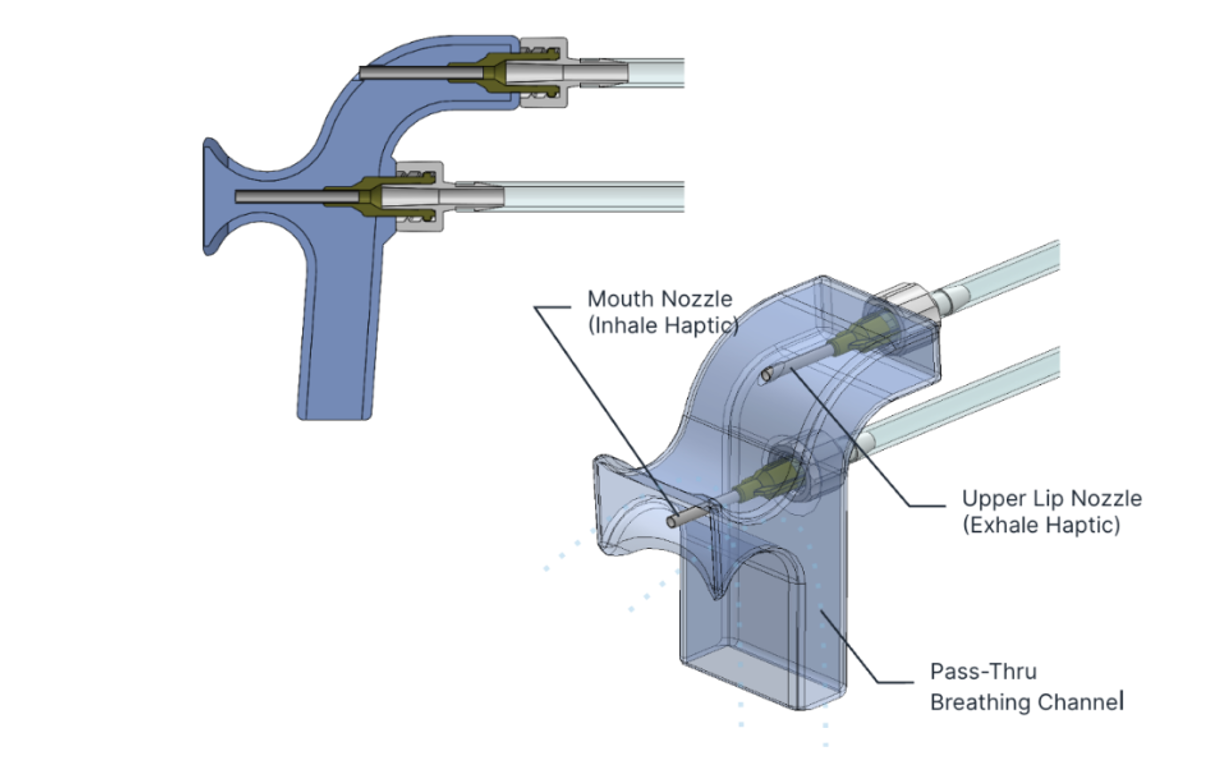

Supplement: Multimedia Appendix 1 [file formative_v10i1e85321_app1.docx]
